# Proteome Analysis of USP7 Substrates Revealed Its Role in Melanoma Through PI3K/Akt/FOXO and AMPK Pathways

**DOI:** 10.3389/fonc.2021.650165

**Published:** 2021-03-31

**Authors:** Lanyang Gao, Danli Zhu, Qin Wang, Zheng Bao, Shigang Yin, Huiyan Qiang, Heinrich Wieland, Jinyue Zhang, Alexander Teichmann, Jing Jia

**Affiliations:** ^1^ Sichuan Provincial Center for Gynaecology and Breast Disease, The Affiliated Hospital of Southwest Medical University, Luzhou, China; ^2^ Academician (Expert) Workstation of Sichuan Province, The Affiliated Hospital of Southwest Medical University, Luzhou, China; ^3^ Laboratory of Nervous System Disease and Brain Functions, The Affiliated Hospital of Southwest Medical University, Luzhou, China; ^4^ Department of Outpatient, The Affiliated Hospital of Southwest Medical University, Luzhou, China; ^5^ Department of Anesthesiology, The Affiliated Hospital of Southwest Medical University, Luzhou, China; ^6^ Laboratory of Anesthesiology, Southwest Medical University, Luzhou, China

**Keywords:** melanoma, USP7, deubiquitinating enzyme, quantitative proteomics, PI3K/Akt/FOXO pathways

## Abstract

The ubiquitin-specific protease 7 (USP7), as a deubiquitinating enzyme, plays an important role in tumor progression by various mechanisms and serves as a potential therapeutic target. However, the functional role of USP7 in melanoma remains elusive. Here, we found that USP7 is overexpressed in human melanoma by tissue microarray. We performed TMT-based quantitative proteomic analysis to evaluate the A375 human melanoma cells treated with siRNA of USP7. Our data revealed specific proteins as well as multiple pathways and processes that are impacted by USP7. We found that the phosphatidylinositol-3-kinases/Akt (PI3K-Akt), forkhead box O (FOXO), and AMP-activated protein kinase (AMPK) signaling pathways may be closely related to USP7 expression in melanoma. Moreover, knockdown of USP7 in A375 cells, particularly USP7 knockout using CRISPR-Cas9, verified that USP7 regulates cell proliferation *in vivo* and *in vitro*. The results showed that inhibition of USP7 increases expression of the AMPK beta (PRKAB1), caspase 7(CASP7), and protein phosphatase 2 subunit B R3 isoform (PPP2R3A), while attenuating expression of C subunit of vacuolar ATPase (ATP6V0C), and peroxisomal biogenesis factor 11 beta (PEX11B). In summary, these findings reveal an important role of USP7 in regulating melanoma progression *via* PI3K/Akt/FOXO and AMPK signaling pathways and implicate USP7 as an attractive anticancer target for melanoma.

## Introduction

Malignant melanomas are an extremely aggressive skin cancer induced by ultraviolet (UV) radiation that arises from melanocytes (1). The global prevalence of melanomas has been growing at an alarming rate over the last several years (2). Until now, there has been no effective therapy for melanoma due to its metastatic potential. Therefore, it is crucial to explore the molecular mechanisms of melanoma progression.

USP7, as a deubiquitinating enzyme (DUB), participates in regulating many cellular process, including tumor progression (3, 4), immune dysfunction (5), DNA damage (6), and epigenetic regulation (7). Among these processes, the roles of USP7 in tumor progression have been extensively characterized as either p53-dependent or p53-independent. Under normal conditions, USP7 prefers to associate with murine double minute2 (MDM2), a E3 ubiquitin ligase targeting p53 for degradation by the proteasome, and stabilizes MDM2, resulting in p53 turnover (8, 9). However, upon cellular stress, USP7 switches from stabilizing MDM2 to p53 (8). Beyond the USP7-MDM2-p53 axis, USP7 also affects oncogenesis through other modulators, including regulation of oncoproteins (such as REST, TRRAP, and cMyc) (10, 11), PTEN (12), FOXO proteins (13), and the Rb protein (14). Therefore, in the past decades, researchers have explored its effects on tissues including the bladder (15), prostate (12), colon (16), lung (17), liver (18), ovary (19), brain (14), breast (20), and glioma (21). All these studies demonstrated that the role of USP7 is tumor suppressive or oncogenic, depending on the context of the cancers and its substrates. However, the expression and role of USP7 in melanoma remains to be elucidated.

We confirmed that USP7 was highly expressed in clinical melanoma tissues and its loss of function significantly inhibited proliferation of melanoma cells and promoted apoptosis. In this study, mass spectrometry-based deep proteomic analysis was carried out following USP7 knockdown to explore the mechanism of action of USP7 in regulating melanoma growth. Our results revealed that USP7 acts as an oncogene in melanoma through mediating PI3K/AKT/FOXO as well as AMPK signaling pathways, and through some new pathways, such as peroxisome and lysosome signaling pathways. Concomitantly, we found ATP6V0C and PEX11B may be new substrates of USP7, which requires further confirmation. In summary, these data suggest that USP7 is a tumor promoter and can serve as a therapeutic target for melanoma, potentially as a novel therapeutic strategy to respond to the resistance of advanced melanoma to chemotherapy.

## Materials and Methods

### Tissue Microarray and TCGA Analysis

The tissue microarray (ME241b) was purchased from Alenabio (Xian, Shanxi, China). This microarray have total 21 tissue sample, including normal skin tissue, melanoma from skin, esophagus, parotid, anus and rectum. All experiments were performed following the protocols and ethical standards of this company. Anti-USP7 (Santa Cruz, 1:50) was used to immunolabel the paraffin-embedded sections. USP7 expression in tissues was evaluated. Kaplan–Meier survival analyses for The Cancer Genome Atlas (TCGA) dataset was conducted using the online database (www.oncolnc.org). P values were calculated with log-rank test. Patients were stratified into “low” and “high” expression based on autoselect best cutoff in the database.

### Immunohistochemical Staining and Intensity Analysis

Immunohistochemistry was performed as previously described (22). The tissues sections were blocked by goat serum and incubated with primary antibody (1:50) and then incubated with biotinylated secondary antibody (1:100). Finally, the data were analyzed with ImageJ software.

### Cell Lines and Cell Culture

A375 human melanoma cells, mouse B16 melanoma cells, and 293T cells were cultured in Dulbecco’s modified Eagle’s medium (HyClone) with high glucose (4.5 g/l) containing 100 IU/ml penicillin/streptomycin and 10% fetal bovine serum (Gibco). All of the cells were cultured at 37°C with 5% CO_2_.

### Transfection Assay

For siRNA transfection, siRNAs targeting USP7 and lipofectamine RNAiMax reagent (Invitrogen) were prepared according to the manufacturer’s instructions. The siRNA sequences were as follows: si-NC (sense 5’-UUC UCC GAA CGU GUC ACG UTT-3; antisense 5’-ACG UGA CAC GUU CGG AGA ATT-3’) and si-USP7 (sense 5’-GGA CUA UGA CGU GUC UCU UTT-3’; antisense 5’-AAG AGA CAC GUC AUA GUC CTT-3’).

### Stable A375 USP7-ShRNA Knockdown Establishment

Sequences of shRNAs are shown as following: ShRNA-NC: 5’-CCTAAGGTTAAGTCGCCCTCG-3’; ShRNA-USP7: 5’-CGTGGTGTCAAGGTGTACTAA-3’.

pCMV-dR8.2 dvpr, pLP/VSVG, and lentiviral DNA constructs (pLKO.1-TRC for USP7) were co-transfected into 293T cells using LipoFilter (HANBIO). Supernatant was collected after 24 h transfection then added to infect A375 cells, cells were selected with puromycin (1 ug/ml) over 7 days.

### Stable B16 CAS9-USP7 Knockout Establishment

USP7 CRISPR/Cas9 plasmid was purchased from Santa Cruz and transfected into B16 cells. After 48 h transfection, B16 cells were cultured into 96-well plates at a concentration of 1 cell/well. Single colonies were detected by immunoblotting analysis. USP7 knockout B16 cells and control cells were defined as B16 USP7 KO and B16 WT, respectively.

### Western Blot Analysis

Total cellular proteins were obtained and denatured, and the A375 nucleus and cytoplasm proteins were obtained by using the Nucleus and Cytoplasmic Protein Extraction Kit (Sangon Biotech), separated by sodium dodecylsulfate polyacrylamide gel electrophoresis (SDS–PAGE) and transferred to nitrocellulose filter membranes NC membranes. The antibodies used are shown in **Table 1**.

**Table 1:** List of All Antibodies and Sources.

antibody	product code	company	host species	dilution
β-Actin	BS6007M	Bioworld	mouse	1:5000
Usp7	sc-377147	Santa Cruz	mouse	1:400
Casp7	A1524	Abclonal	rabbit	1:1000
ATP6V0C	A16350	Abclonal	rabbit	1:500
PRKAB1	A12491	Abclonal	rabbit	1:1000
PEX11B	A18321	Abclonal	rabbit	1:500
PARP1	A19596	Abclonal	rabbit	1:500
Phospho-FOXO4-S197	AP0177	Abclonal	rabbit	1:1000
FOXO4	A3307	Abclonal	rabbit	1:500
PPP2R3A	A17395	Abclonal	rabbit	1:500
AKT	#9272	Cell Signaling	rabbit	1:1000
P-Akt1(Ser473)	AF1546	Beyotime	rabbit	1:600
p-FOX01(Thr24)/Fox03a(Thr32)/Fox04(Thr28)	#2599	Cell Signaling	rabbit	1:1000
P-AMPKB1-S108	AP0597	Abclonal	rabbit	1:600
Ki-67	Sc-23900	Santa Cruz	mouse	1:200
P53	#2524	Cell Signaling	mouse	1.1000
MDM2	Sc-965	Santa Cruz	mouse	1:500
Rabbit IgG Mouse IgG	AS014 AS003	Abclonal Abclonal	— —	1:20000 1:20000

#### Cell Clonogenic Assay

A clonogenic assay was performed as previously described (23). Wild-type cells and USP7 loss cells (A375 shRNA-USP7 and B16 WT/USP7-KO) were plated into six well plates in triplicate. Then wild-type cells were exposed to the USP7 inhibitor gen6776 (10 μm). The plates were washed with PBS and stained with crystal violet on the 14^th^ day. The cells were observed and photographed.

### Immunofluorescence

A375 cells were transfected with si-NC/USP7. After 24 h, cells were fixed with 4% paraformaldehyde and blocked with 0.2% Triton X-100 and 5% FBS in PBS. Samples were incubated with primary antibody FOXO4 (1:100, Abclonal) and Ki-67 (1:200, Santa Cruz) in PBS overnight at 4 °C. Samples were incubated with secondary FITC-labeled anti-rabbit and Cy3-labeled anti-mouse antibody (Beyotime) for 1 h at ambient temperature. Cells were visualized with an Olympus X71 fluorescence microscope.

### Cell Cycle and Apoptosis Assay

For the cell cycle assay, 1 ml of 70% ethanol was added to cells and cells were resuspended overnight. Then the cells were incubated with 50 μg/ml of propidium iodide (PI), 0.2% of Triton-X-100, and 100 μg/ml of RNase complex for 30 min in the dark. For the cell apoptosis analysis, cells were resuspended for 30 min with a complex of 500 μl Annexin VFITC binding buffer, 5 μl PI, and 5 μl Annexin V-FITC. Finally, the two samples were assessed by flow cytometry.

### Quantitative Reverse Transcription PCR (RT-qPCR) Analysis

Total RNA was extracted by TRIzol reagent (Invitrogen), and HiScript III RT SuperMix for RT-qPCR (Vazyme) was used for reverse transcription according to the manufacturer’s instructions. The primers used for RT-qPCR are shown as follows: p27^Kip1^ (forward): 5’-GGCTAACTCTGAGGACACGCA-3’; p27^Kip1^ (reverse): 5’-TGGGGAACCGTCTGAAACAT-3’; GAPDH (forward): 5’-GGAGCGAGATCCCTCCAAAAT-3’; and GAPDH (reverse): 5’-GGCTGTTGTCATACTTCTCATGG-3’.

### Proteomic Sample Preparation

The siRNA transfected A375 cell samples were disrupted in SDT (4% (w/v)) SDS, 100 mM Tris/HCl (pH7.6, 0.1M DTT) lysis buffer. The protein concentration was determined by BCA (bicinchoninic acid) assay.

Tandem mass tag (TMT) labeling and high pH reversed-phase peptide fractionation and LC-MS/MS analysis. Proteomic samples were analyzed by LC-MS/MS as described in **Supplement 1**.

### Protein Identification and Quantitation

LC**-**MS/MS spectra were searched using the MASCOT engine (Matrix Science, London, UK; version 2.2) embedded into Proteome Discoverer 1.4 (Thermo Scientific). The protein ratios were calculated as the median of only unique peptides of the protein. All peptide ratios were normalized by the median protein ratio. The median protein ratio should be 1 after the normalization.

### Animal Experiments

Nude female mice (Balb/c) and C57BL/6 female mice were purchased from the Chengdu Dossy Laboratory Animals Company. All of the animal procedures were approved by the Laboratory Animal Management Committee of the Affiliated Hospital of Southwest Medical University. The animals were treated as described in **Supplement 2**.

### Statistical Analysis

The data shown in the figures are representative of three or more independent experiments and were analyzed by the one-way Student’s *t*-test, and P < 0.05 was considered statistically significant. Where exact P-values are not shown, statistical significance is shown as *P < 0.05, **P < 0.01.

## Results

### USP7 Is Overexpressed in Melanoma and Predicts Clinical Outcomes

**Figure 1:**
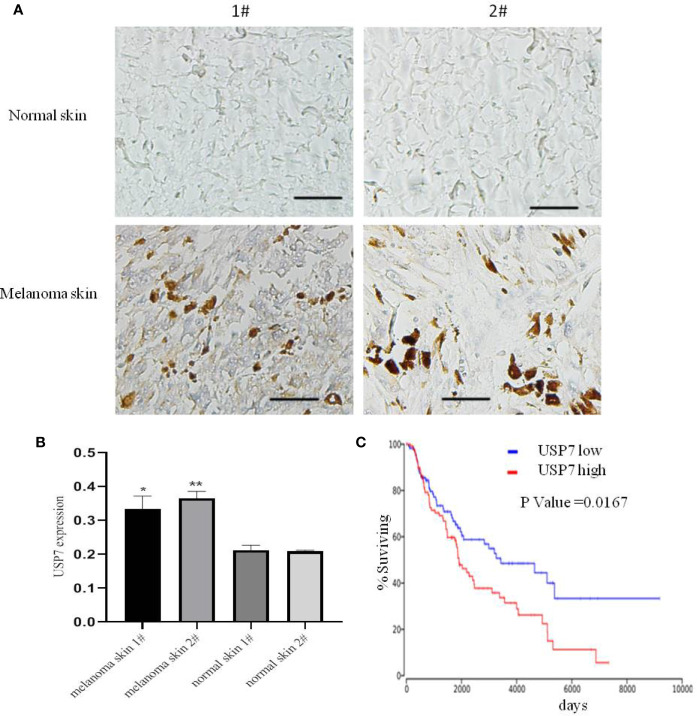
USP7 is overexpressed in melanoma and predicts clinical outcome. **(A, B)** Immunohistochemical analysis of USP7 in human normal skin and melanoma tissue. The data were analyzed with ImageJ software. Scale bar, 50 μm. *p<0.05,**p<0.01. **(C)** Kaplan-Meier curves from patients with melanoma expressing low and high USP7 from the TCGA protein expression array data.

Previous studies have demonstrated that USP7 plays a prominent role in tumor development and progression (12, 14, 18). We found that the expression of the USP7 protein was increased in melanoma tissues compared with normal tissue by immunohistochemistry (**Figures 1A, B**). Furthermore, we performed Kaplan–Meier analysis in melanoma patients of TCGA. The results showed that USP7 expression was negatively correlated with overall survival of melanoma patients in the TCGA dataset (P < 0.05, **Figure 1C**). Collectively, these results demonstrate that USP7 is overexpressed in human melanoma and predicts clinical outcomes.

### USP7 Loss Suppresses Melanoma Growth

**Figure 2:**
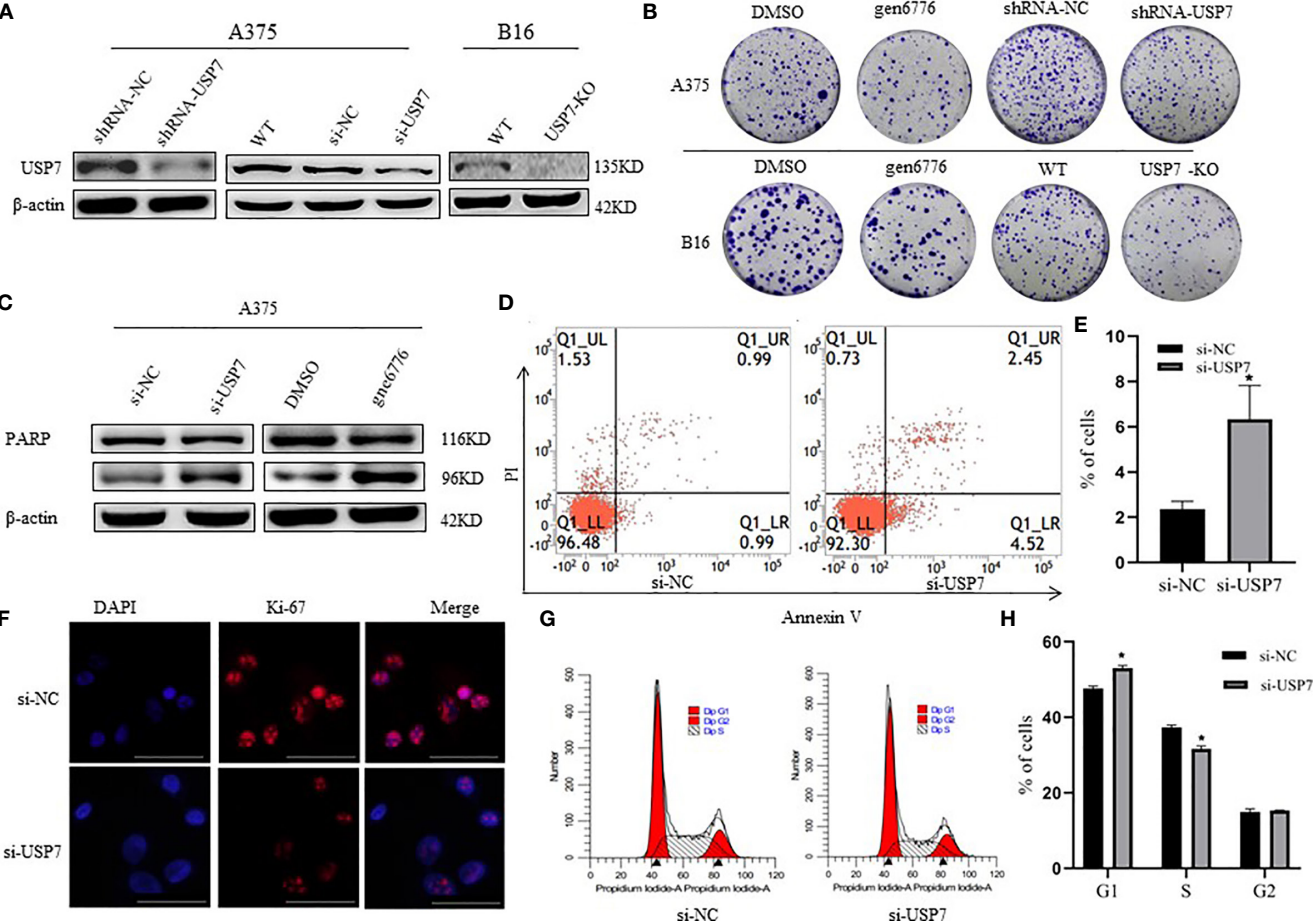
Effects of USP7 on melanoma cell line growth. **(A, B)** Protein lysates were extracted from A375 cells transiently expressing USP7 siRNA and stable control shRNA or USP7 shRNA and B16 cells deleted the USP7 gene using the CRISPR/Cas9 editing system (USP7 KO). Western blotting analysis was performed to determine the expression of USP7. **(B)** Cells were collected from the indicated cells with gen6776 (USP7 inhibitor), USP7 shRNA or USP7 knockout treatment. Colony formation assay was performed and representative images are shown. **(C–E)** A375 cells were transfected with USP7 siRNAs for 48 h and detected with Annexin V-FITC/PI staining followed by flow cytometry analysis. Cell death populations are shown. Western blotting analysis was used for PARP expression. **(F–H)** A375 cells were treated as described in **(C–E)**. Immunofluorescence staining of Ki-67 of A375 cells was observed using fluorescence microscope. Red: Ki-67; blue: nucleus. Typical images are shown. Scale bars: 50 μm. Analysis of cell cycle by FCM is described in the *Materials and Methods*. Data are represented as the mean ± SEM of three independent experiments, each in triplicate; bars, SEM. *P ≤ 0.05 vs. control.

To explore USP7 expression levels relative to melanoma development and progression, we built USP7 knockdown A375 cells by shRNA or siRNA and USP7 KO B16 cells (**Figure 2A**). The cell colony formation assay results revealed that loss of USP7 function dramatically suppressed colony formation (**Figure 2B**). Furthermore, **Figures 2C–E** showed that poly ADP-ribose polymerase (PARP) cleavage and cellular apoptosis were induced by USP7 inhibition. Concomitantly, USP7 downregulation significantly arrested the cell cycle, increasing the proportion of A375 cells in the G1 phase and decreasing the proportion in the S phase (**Figures 2G, H**). Expression of the proliferation marker Ki67 was also reduced by USP7 downregulation (**Figure 2F**). These findings support a role for USP7 in promoting growth of melanoma cells.

### Protein Identification and Bioinformatics Analysis

To clarify the mechanism of action of USP7 in melanoma, we compared whole-cell proteomes in WT and USP7 knockdown A375 cells using TMT quantitative proteomics technology. As a result, we identified a total of 5696 proteins with expression changes of 1.1 times or more and P values of < 0.05. In the si-USP7 vs si-NC group, we found 101 upregulated differentially expressed proteins (DEPs) and 71 downregulated DEPs (**Supplement 4**). **Supplement 1B** shows a hierarchical clustering analysis of all 172 DEPs.

**Figure 3:**
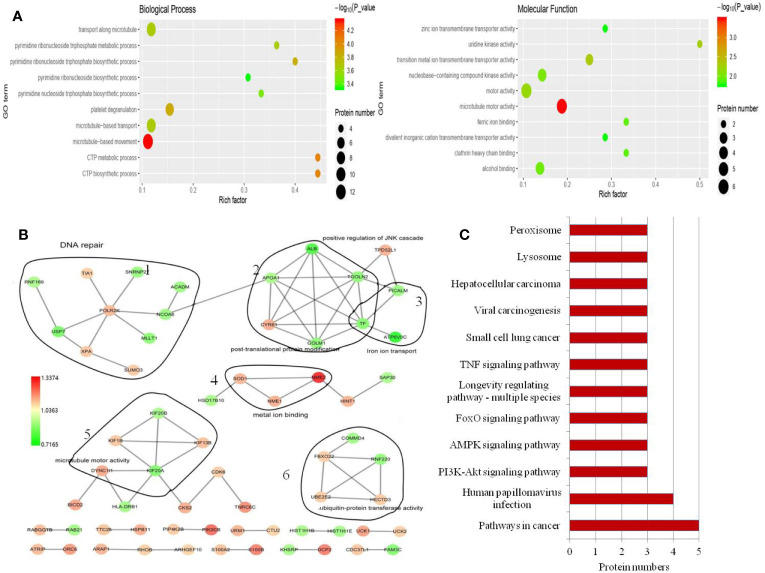
GO annotation and KEGG pathway analysis of the DEPs. **(A)** Top 10 enriched GO terms under “biological process”. Term01, microtubule-based movement; term02, CTP metabolic process; term03, CTP biosynthetic process; term04, platelet degranulation; term05, pyrimidine ribonucleoside triphosphate biosynthetic process; term06, microtubule-based transport; term07, transport along microtubule; term08, pyrimidine ribonucleoside triphosphate metabolic process; term09, pyrimidine nucleoside triphosphate biosynthetic process; term10, pyrimidine ribonucleoside biosynthetic process. Top 10 enriched GO terms under “molecular function”. Term01, microtubule motor activity; term02, transition metal ion transmembrane transporter activity; term03, uridine kinase activity; term04, motor activity; term05, nucleobase-containing compound kinase activity; term06, alcohol binding; term07, clathrin heavy chain binding; term08, ferric iron binding; term09, zinc ion transmembrane transporter activity; term10, divalent inorganic cation transmembrane transporter activity. **(B)** Protein–protein interaction network generated with STRING and visualized with Cytoscape for DEPs. DEPs are represented as round nodes. The red node indicates upregulation and green node indicates downregulation of the DEPs. Corresponding to proteins related to DNA repair (1), post-translational protein modification and iron ion transport (2), microtubule motor activity (3), metal ion binding (4) and ubiquitin-protein transferase activity (5), ubiquitin-protein transferase activity (6). **(C)** Enriched KEGG pathways.

A total of 5696 proteins were annotated to molecular function, biological process, and cellular components by GO analysis. Then, the Fisher’s exact test was used to analyze the GO function enrichment of the DEPs. The top 10 enriched GO terms within each major functional category are shown in **Figure 3A** and **Supplement 1C**. In the three categories, the most significant terms are all associated with microtubule-related functions (**Figure 3C**).

**Figure 4:**
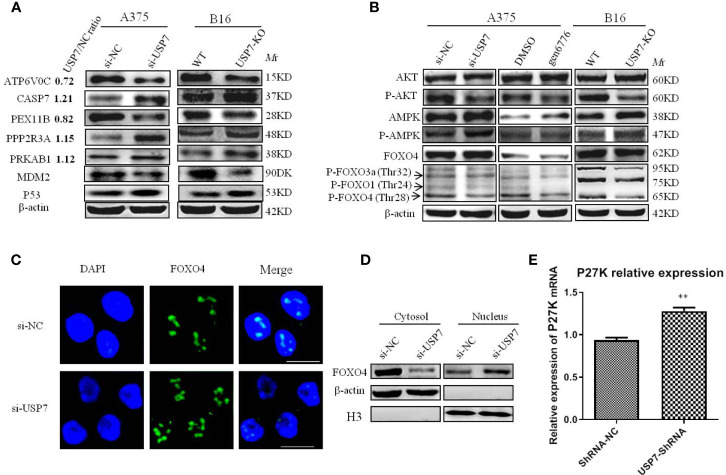
Multiple proteins and PI3K/AKT/FOXO and AMPK signaling pathways are affected by removal of USP7. USP7 was inhibited by siRNA or inhibitor gen 6776 in A375 cells and was knocked out through CRISPR/Cas9 in B16 cells. **(A)** Proteins exhibiting significant changes between USP7 knockdown A375 cells and A375 cells and USP7-KO B16 cells. Protein names, USP7/NC ratio, and the expression of indicated proteins by western blotting are shown. **(B)** The key proteins levels of the PI3K/Akt/FOXO and AMPK signaling pathways were analyzed by western blotting after inactivation of USP7 in A375 cells and B16 cells. **(C, D)** The expression of FOXO4 in the nucleus and cytosol was detected by immunofluorescence and western blotting, respectively. Green, FOXO4; blue, nucleus. Scale bar 50 μm. **(E)** Representative levels of P27^kip1^ by quantitative real-time PCR. Data are represented as the mean ± SEM of three independent experiments, each in triplicate; bars, SEM. **P ≤ 0.01 vs. control.

In the biological process and molecular function category, we noted that many terms are related to nucleoside-containing compound biosynthesis and metabolism (**Figure 3A**). We also identified significantly altered protein-protein interaction networks in the protein sets, as shown in **Figure 3B**. Among proteins involved in ion transport and binding (**Figure 3B**), ATP6V0C had the most decreased protein expression following USP7 knockdown, which was also confirmed by western blot (**Figure 4A**). Protein-protein interaction networks then revealed another four distinct clusters (**Figure 3B**), corresponding to proteins related to DNA repair, post-translational protein modification, microtubule motor activity, and ubiquitin-protein transferase activity.

To explore how USP7 plays roles in melanoma by these cellular processes, DEPs were then mapped to the reference pathway in the KEGG database. There were 172 DEPs that were mapped to 178 signaling pathways. Phosphatidylinositol 4,5-bisphosphate 3-kinase catalytic subunit beta isoform (PIK3CB) is the protein that most commonly participates in signaling pathways, followed by PRKAB1, CASP7, PPP2R3A, and ATP6V0C. We confirmed these proteins in both A375 and B16 cell lines (**Figure 4A**). Concomitantly, only the pathways with at least three or more DEPs are shown in **Figure 3C**. In this study, we mainly focused on the PI3K-Akt, FOXO, and AMPK signaling pathways to clarify the roles of USP7 in melanoma.

### Validation of Proteomic Data

To validate our mass spectrometry results, we investigated the most striking DEPs including ATP6V0C and PEX11B, PRKAB1 (AMPK), CASP7, and PPP2R3A, which participate in many pathways (**Figure 4A**). Altogether, these proteins were confirmed in both melanoma cell lines after USP7 loss, which indicated that USP7 may control the signaling pathways involving in these proteins to promote melanoma development and progression. For example, Kaplan–Meier analysis of ATP6V0C and CASP7 in melanoma patients of TCGA showed that expression of the two proteins was negatively and positively correlated with overall survival of melanoma patients (**Supplement 5**), respectively.

### Reduction of USP7 Mediates Signaling Pathways Associated With Cancer Activity

Immunoblotting results showed that phosphorylated AMPK was significantly increased following USP7 loss in both cell lines A375 and B16 (**Figure 4B**), which may be due to an increase in PRKAB1. Previous studies have proposed a tumor-suppressing function of activated AMPK (24, 25). Accordingly, USP7 loss inhibits melanoma growth by partially activating the AMPK signaling pathway.

Consistent with previous studies on USP7 (12, 13, 26), our results suggest that USP7 can activate the PI3K/Akt signaling pathway to promote cell survival by phosphorylating and inhibiting FOXO transcription factors, such as FOXO1, FOXO3a, and FOXO4. As shown in **Figures 4A** and **B**, phosphorylated Akt decreases due to the increase in PPP2R3A, which can dephosphorylate Akt. Further research has found that Akt phosphorylation reduction results in FOXO dephosphorylation at Akt-induced sites, including P-FOXO4(Thr28), P-FOXO3a(Thr32), and P-FOXO1(Thr24) (**Figure 4B**). Previous studies have indicated that a decrease in FOXO phosphorylation promotes their entry into the nucleus and ultimately increases transcriptional activity towards target genes, including the cell cycle arrest gene p27^kip1^ (26). To further confirm this result in melanoma, FOXO4 was selected for detecting its intracellular localization by immunofluorescence and nuclear/cytosol protein fractionation assay and its target gene p27^kip1^ expression levels. Our results showed that FOXO4 was mainly present in the nuclear compartment after USP7 downregulation (**Figures 4C, D**), which ultimately promoted p27^kip1^ expression (**Figure 4E**).

Taken together, our results suggest that loss of USP7 function inhibits the melanoma cell cycle and promotes cell apoptosis by mediating AMPK and PI3K/Akt/FOXO signaling pathway activity. The results also demonstrated that USP7 can upregulate MDM2, and inhibit the expression of p53.

### USP7 Loss Suppresses Melanoma Growth in A375 and B16 Xenografts

**Figure 5:**
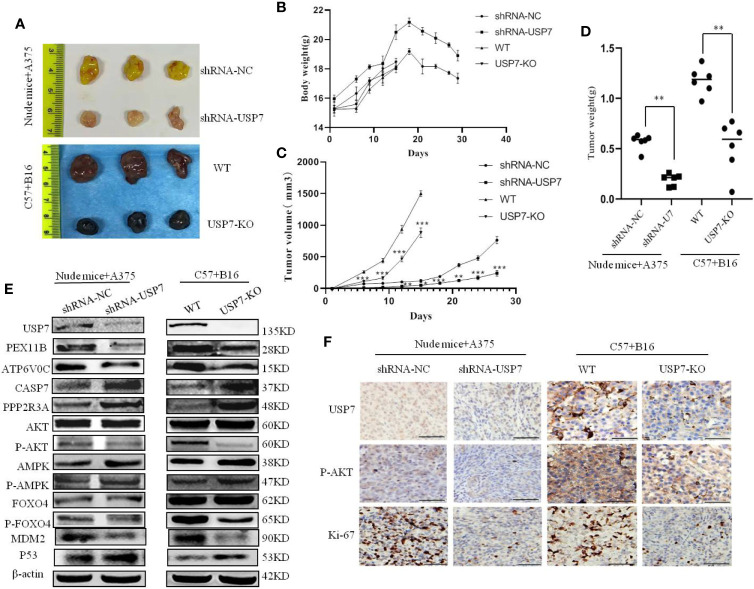
Loss of USP7 function suppresses melanoma tumor growth *in vivo*. Balb/c nude mice and C57BL/6 mice were subcutaneously transplanted with A375 cells stably expressing USP7 shRNA and USP7-KO B16 cells, respectively, as indicated in the *Materials and Methods*. **(A)** Images of harvested mice and tumors after treatment from A375 shRNA-NC cells, A375 shRNA-USP7 cells, B16 WT melanoma cells, or B16 USP7-KO melanoma cells. **(B)** Mouse weight evolution during the experiment. The values are expressed as the mean. **(C)** Tumor size was determined by caliper measurement and the data was converted to tumor growth curves. **(D)** Tumor tissues were harvested and weighed at the end of study (**P < 0.01. Error bar = S.D.). **(E, F)** Expression of indicated proteins in tumor tissues was measured by western blotting and immunohistochemistry.

ShRNA-USP7 A375 cell and USP7-KO B16 cell xenograft models were established in Balb/c nude mice and C57BL/6 mice, respectively. As shown in **Figures 5A–C**, there was a significant reduction in tumor growth and tumor weight after USP7 loss, with tumor volumes inhibited by 64.7% for A375 and 46.4% for B16. No significant change was observed in the body weight between the control and USP7 loss groups.

To further validate the mechanism of action of USP7 as an oncogene in melanoma *in vivo*, the expression levels of the proteins indicated in **Figure 5** were analyzed by immunoblotting and immunohistochemistry. **Figures 5E** (The Western blot analysis of proteins in mice tumors were evaluated **Supplement 6**) and **5F** show that in line with the proteomic results, the indicated proteins have significant changes on levels in both A375 and B16 cells after USP7 loss.

## Discussion

We proved that USP7 levels are higher in melanoma than that of normal skin and are associated with poorer prognosis based on analysis from TCGA datasets. These findings implied that USP7 is implicated in melanoma development and progression. In this study, we demonstrated that loss of USP7 function inhibits cell growth by promoting cell cycle arrest and apoptosis in A375 and B16 cells. Furthermore, proteomic data analysis and western blot results showed the importance of the classical signaling pathways PI3K/Akt/FOXO and AMPK, and that new biological processes involve proteins such as ATP6V0C, CASP7, and PEX11B, which play crucial roles in USP7 mediating melanoma growth. USP7 knockdown decreases Akt phosphorylation and then causes FOXO phosphorylation reduction, which ultimately increases FOXO accumulation in the nucleus and promotes P27^kip1^ gene expression that arrests the cell cycle (**Figure 4**).

In addition to our studies, several groups have shown that there are two factors of Akt phosphorylation reduction that must be addressed. The first involves PPP2R3A, a regulatory subunit of the protein phosphatase 2 (PP2A), which is significantly increased after USP7 downregulation. A previous study has shown that PP2A can directly dephosphorylate Akt, inhibiting PI3K/Akt signaling pathway activity (27). The second relates to the intracellular localization of PTEN, which antagonizes the PI3K-AKT pathway. Previous reports have demonstrated that USP7 induces PTEN deubiquitination, causing exclusion of PTEN from the nucleus and subsequently increasing in the PI3K/AKT signaling pathway (28). Therefore, USP7 downregulation accumulates monoubiquitinated PTEN in the nucleus, reducing Akt phosphorylation. Collectively, these two factors explain how USP7 plays a role in melanoma through the PI3K-AKT pathway.

Further research has shown that Akt phosphorylation reduction induces FOXO dephosphorylation at Akt-induced sites. It has been reported that phosphorylated FOXO induced by activated Akt stays in the cytosol and is imported to the nucleus through dephosphorylation to induce the expression of a series of target proteins that regulate metabolism, the cell cycle, and apoptosis (29). Our results indicate that phosphorylation of FOXO4 (Thr28), FOXO3a (Thr32), and FOXO1 (Thr24) significantly decreases and FOXO4 substantially accumulates in the nucleus following USP7 knockdown. FOXO4 accumulation in the nucleus may be caused by weak deubiquitination of USP7 downregulation. Similar to PTEN, USP7-induced FOXO4 deubiquitination results in nuclear export and eliminates its transcriptional activity (13).

KEGG analysis of the proteomic data and western blot results reveal that the AMPK signaling pathway plays a role in USP7 mediating melanoma growth. PRKAB1, a regulatory subunit of AMPK, has a remarkable up-regulation following loss of USP7 function, which ultimately raises AMPK phosphorylation and activates AMPK signaling. It is well-documented that AMPK signaling has an anti-proliferative role. Dai et al. have reported that activated AMPK signaling inhibits survival and proliferation and activates apoptosis in colorectal cancer cells (30). Consequently, we conclude that USP7 plays a role in melanoma through AMPK signaling.

USP7 as a context-specific modulator mediates p53-dependent apoptosis *via* controlling MDM2 stability. Inhibition of USP7 also induces endoplasmic reticulum (ER) stress due to the accumulation of polyubiquitinated protein substrates in cancer cells, which leads to increased intracellular reactive oxygen species (ROS). Increased ROS can cause apoptosis in these cells. Our results indicated that USP7 knockdown also induces apoptosis (**Figure 2**). A lack of USP7-dependent deubiquitylation of MDM2 may lead, through enhanced breakdown of MDM2, to accumulation of p53 in melanoma. However, our data suggests additional involvement of USP7 in alternative apoptotic pathways, possibly *via* modification of a caspase7 dependent mechanism. In line with these observations, we found cleaved PARP1 levels increased upon USP7 removal. Similarly, in colon carcinoma cells, USP7 is involved in apoptotic pathways by modifying caspase3 levels (31). Therefore, we confirm that USP7 also as an oncogene inhibits p53-dependent apoptosis in melanoma, as in colon cancer.

ATP6V0C and PEX11B, proteins regulated by USP7, were confirmed in both melanoma cell lines by western blot. It has been reported that ATP6V0C is involved in the migration and invasion of prostate carcinoma cells (32). In addition, enriched KEGG pathways show that ATP6V0c and PEX11B are key protein of lysosome and peroxisome pathways, respectively. These findings provide a cue that USP7 plays roles in melanoma through these two pathways that have not been reported in other cancers.

Previous results have demonstrated that inhibition of USP7 induces genotoxic stress and DNA damage in chronic lymphocytic leukemia (CLL) cells (33). We also obtained similar results in melanoma cells following USP7 knockdown. GO analysis suggests that the DEPs RNF169, XPA, MLLT1, and NCOA6 participate in DNA repair (**Figure 3**). One of these, E3 ubiquitin ligase RNF169 is deubiquitylated and stabilized by USP7 in response to DNA double-strand breaks (34), and is significantly decreased in the USP7 knockdown, which suggests USP7 in melanoma also plays a role in DNA repair.

## Data Availability Statement

The authors acknowledge that the data presented in this study must be deposited and made publicly available in an acceptable repository, prior to publication. Frontiers cannot accept an article that does not adhere to our open data policies.

## Ethics Statement

The animal study was reviewed and approved by the Laboratory Animal Management Committee of the Affiliated Hospital of Southwest Medical University.

## Author Contributions

JJ and AT designed the study. LG and JZ drafted the manuscript. DZ, JJ, and LG conducted experiments, and the other authors took part in literature collection and data analysis. All authors contributed to the article and approved the submitted version.

## Funding

This work was supported by the Key Fund and the Youth Fund and the Transformation Project of Science and Technology Achievements of Southwest Medical University (2018-ZRQN-014), Scientific research project of the education department of Sichuan province (17ZA0436), Scientific Research Foundation for Doctors of the Affiliated Hospital of Southwest Medical University (to Lanyang Gao, Jing Jia and Shigang Yin), and Special project of talent introduction in Luzhou (0903-00040055).

## Conflict of Interest

The authors declare that the research was conducted in the absence of any commercial or financial relationships that could be construed as a potential conflict of interest.
