# Corrigendum: Proteome Analysis of USP7 Substrates Revealed Its Role in Melanoma Through PI3K/Akt/FOXO and AMPK Pathways

**DOI:** 10.3389/fonc.2021.736438

**Published:** 2021-07-23

**Authors:** Lanyang Gao, Danli Zhu, Qin Wang, Zheng Bao, Shigang Yin, Huiyan Qiang, Heinrich Wieland, Jinyue Zhang, Alexander Teichmann, Jing Jia

**Affiliations:** ^1^ Sichuan Provincial Center for Gynaecology and Breast Disease, The Affiliated Hospital of Southwest Medical University, Luzhou, China; ^2^ Academician (Expert) Workstation of Sichuan Province, The Affiliated Hospital of Southwest Medical University, Luzhou, China; ^3^ Laboratory of Nervous System Disease and Brain Functions, The Affiliated Hospital of Southwest Medical University, Luzhou, China; ^4^ Department of Outpatient, The Affiliated Hospital of Southwest Medical University, Luzhou, China; ^5^ Department of Anesthesiology, The Affiliated Hospital of Southwest Medical University, Luzhou, China; ^6^ Laboratory of Anesthesiology, Southwest Medical University, Luzhou, China

**Keywords:** melanoma, USP7, deubiquitinating enzyme, quantitative proteomics, PI3K/Akt/FOXO pathways

In the original article, there was a mistake in ***Figure 2C*** as published. In fact, we initially left a gap between 116kD and 96kD WB bands in the manuscript (***Figure 2C***), which might not be clear enough, eventually leading to a misunderstanding of the bands. Actually, the PARP antibody (Abclonal, A19596) we used in this study could detect the full length PARP (116kDa), as well as the cleaved PARP (96 kDa). The short time exposure showed an increased but fuzzy band of the cleaved PARP. As the PARP cleavage serves as a better indicator of apoptosis triggering, that we examine the cleaved PARP (96 kDa) with a long time exposure. The corrected ***Figure 2C*** appears below.

**Figure 2:**
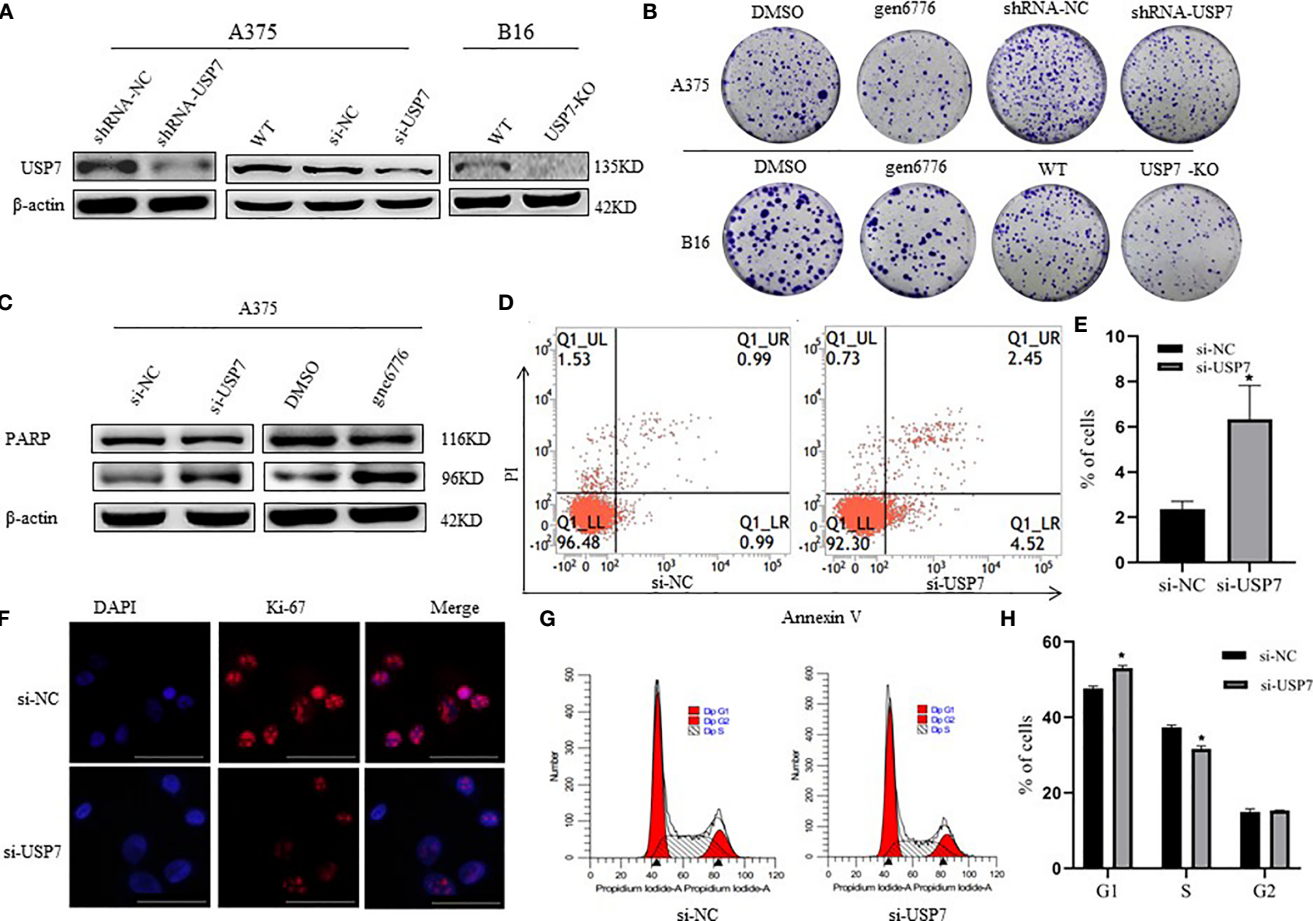
Effects of USP7 on melanoma cell line growth. **(A, B)** Protein lysates were extracted from A375 cells transiently expressing USP7 siRNA and stable control shRNA or USP7 shRNA and B16 cells deleted the USP7 gene using the CRISPR/Cas9 editing system (USP7 KO). Western blotting analysis was performed to determine the expression of USP7. **(B)** Cells were collected from the indicated cells with gen6776 (USP7 inhibitor), USP7 shRNA or USP7 knockout treatment. Colony formation assay was performed and representative images are shown. **(C–E)** A375 cells were transfected with USP7 siRNAs for 48 h and detected with Annexin V-FITC/PI staining followed by flow cytometry analysis. Cell death populations are shown. Western blotting analysis was used for PARP expression. **(F–H)** A375 cells were treated as described in **(C–E)**. Immunofluorescence staining of Ki-67 of A375 cells was observed using fluorescence microscope. Red: Ki-67; blue: nucleus. Typical images are shown. Scale bars: 50 μm. Analysis of cell cycle by FCM is described in the Materials and Methods. Data are represented as the mean ± SEM of three independent experiments, each in triplicate; bars, SEM. *P ≤ 0.05 vs. control.

The authors apologize for this error and state that this does not change the scientific conclusions of the article in any way. The original article has been updated.

## Publisher’s Note

All claims expressed in this article are solely those of the authors and do not necessarily represent those of their affiliated organizations, or those of the publisher, the editors and the reviewers. Any product that may be evaluated in this article, or claim that may be made by its manufacturer, is not guaranteed or endorsed by the publisher.

