# Supplementary material for: Proteome Analysis of USP7 Substrates Revealed Its Role in Melanoma Through PI3K/Akt/FOXO and AMPK Pathways
Source: Front Oncol. 2021 Mar 31;11:650165. doi: 10.3389/fonc.2021.650165 (PMC8044529; doi:10.3389/fonc.2021.650165)

Supplement 1

Tandem mass tag (TMT) labeling and High pH Reversed-Phase Peptide Fractionation and LC-MS/MS analysis

The equal amounts of protein from each group were Trypsin digestion by Filter aided proteome preparation (FASP) method and peptide concentration was determined using absorbance values at 280 nm. 100 μg of each group samples were respectively labeled using the TMT Kit (Thermo) according to the manufacturer's instructions. The labeled peptides in each group were mixed in equal amounts, and separated into fractions according to the High pH Reversed-Phase Peptide Fractionation Kit. Each fraction was dried, dissolved in 0.1% FA (0.1% formic acid, 5% acetonitrile), then, determined the peptide concentration at 280 nm. Each fraction sample were separated by HPLC system. The column was balanced with 95% liquid A(0.1%Aqueous formic acid), then samples flowing through the column (Thermo Scientific Acclaim PepMap100, 100 μm*2 cm, nanoViper C18) and the column (Thermo scientific EASY column, 10cm, ID75 μm, 3 μm, C18-A2) at a rate of 300 nL/min. the Q-Exactive mass spectrometer was used for detection of each fraction after separation. MS data were acquired from 300-1800 m/z for fragmentation by higher-energy collisional dissociation (HCD), with up to 20 precursors selected for LC-MS/MS and dynamic exclusion for 60s. Survey scans were acquired at a resolution of 70,000 at m/z 200 and resolution for HCD spectra was set to 17,500 at m/z 200. Full MS automatic gain control (AGC) target was 1e6, Maximum IT was 50ms and isolationwindow was 2 m/z. The normalized collision energy was 30 eV and the underfill ratio was definedas 0.1%.

Supplement 2

Animal experiments

Nude female mice (body weight 14-16g, 5 weeks old) were randomly divided into two group (six mice per group). Mice were inoculated subcutaneously in the right armpit with 1x106 A375 shRNA-NC cells or A375 shRNA-USP7 cells in 100 μl PBS to induce the tumor. Weight and tumor size of mice were measured at day 6 post tumor transplantation and every three days. Tumor volume was calculated according to the formula $V=0.5\times a^2\times b$ (a, smallest superficial diameter; b, largest superficial diameter). The mice were killed by CO2 overdose at day 29 post treatments. The tumors were harvested, weighted, and recorded.

C57BL/6 female mice (body weight 13-15g, 5 weeks old) were also randomly divided into two group (six mice per group). B16 WT melonoma cells or B16 USP7-KO melonoma cells were subcutaneously injected into their right armpit with the viable cell number 3x105 cells. Weight and tumor size of mice were measured at day 6 post tumor transplantation and every three days. The mice were killed by CO2 overdose at day 15 post treatments. The tumors were analyzed..

Supplement 3

Immunohistochemical analysis of USP7 in human normal skin and other melanoma tissues. Scale bar, 50 μm.

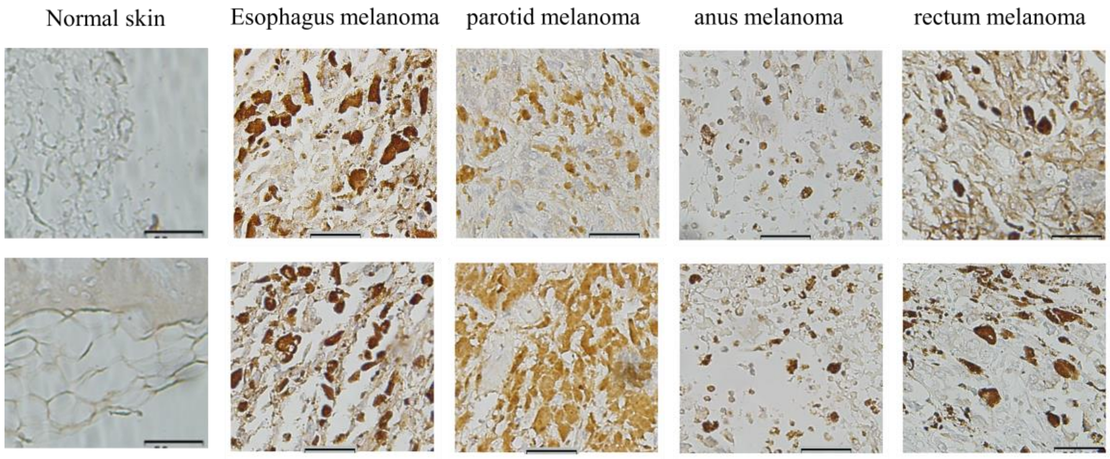

Supplement 4

Integrative analysis of identified proteins. iTRAQ coupled with LC-MS/MS analysis proteins from A375 cells transfected with USP7 siRNA for 24h. (a) The ratio intensity plot representing protein fold and protein density plot. Red, blue and green clusters indicate up-, down- and unregulated proteins, respectively. (b) Heatmap shows the relative changes in abundance of the 172 differentially expressed proteins. (C)Top 10 enriched GO terms "cellular components". term01, kinesin complex; term02, P-body; term03, muscle thin filament tropomyosin; term04, platelet dense granule lumen; term05, axon cytoplasm; term06, intraciliary transport particle B; term07, striated muscle thin filament; term08, cytoplasmic ribonucleoprotein granule; term09, myofilament; term10, microtubule associated complex.

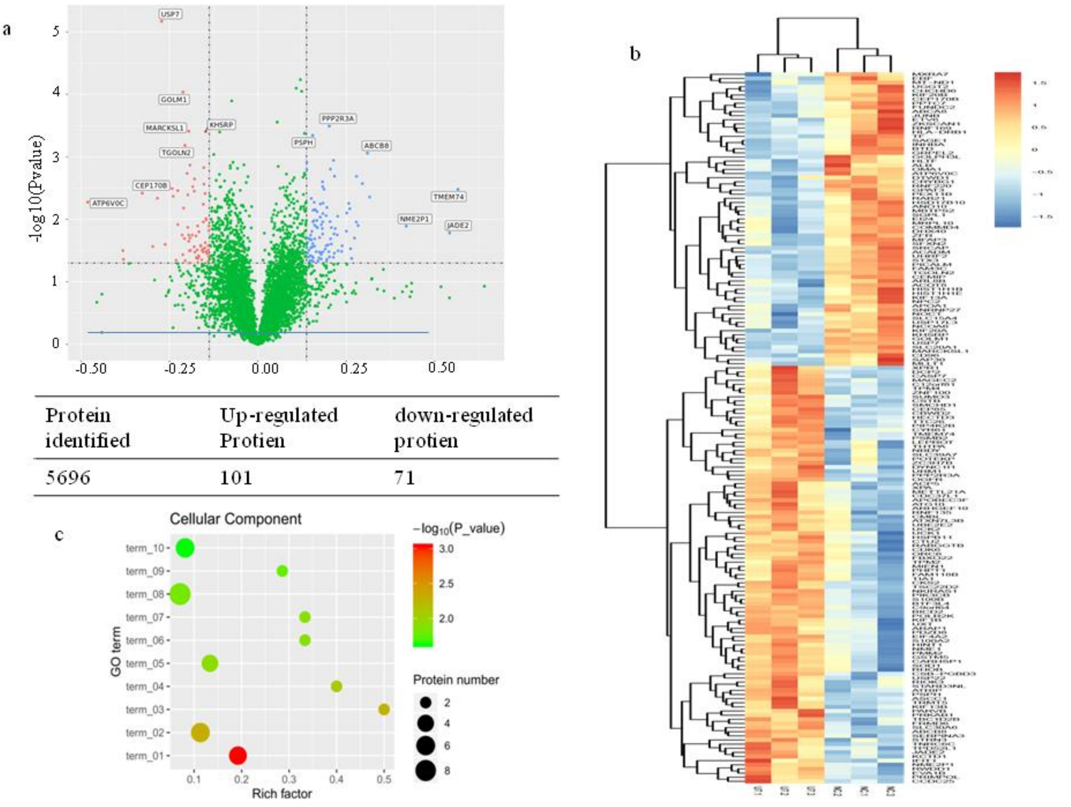

Protein identified	Up-regulated Protien	down-regulated protien
5696	101	71

Supplement 5

Kaplan-Meier curves of ATP6V0C , KIF20A and CASP7 in melanoma patients. (a) Kaplan-Meier curves of the survival between ATP6V0C-high and ATP6V0C–low melanoma patients. (b)

Kaplan-Meier curves of CASP7 in melanoma patients. (c) Kaplan-Meier curves of KIF20A in melanoma patients.

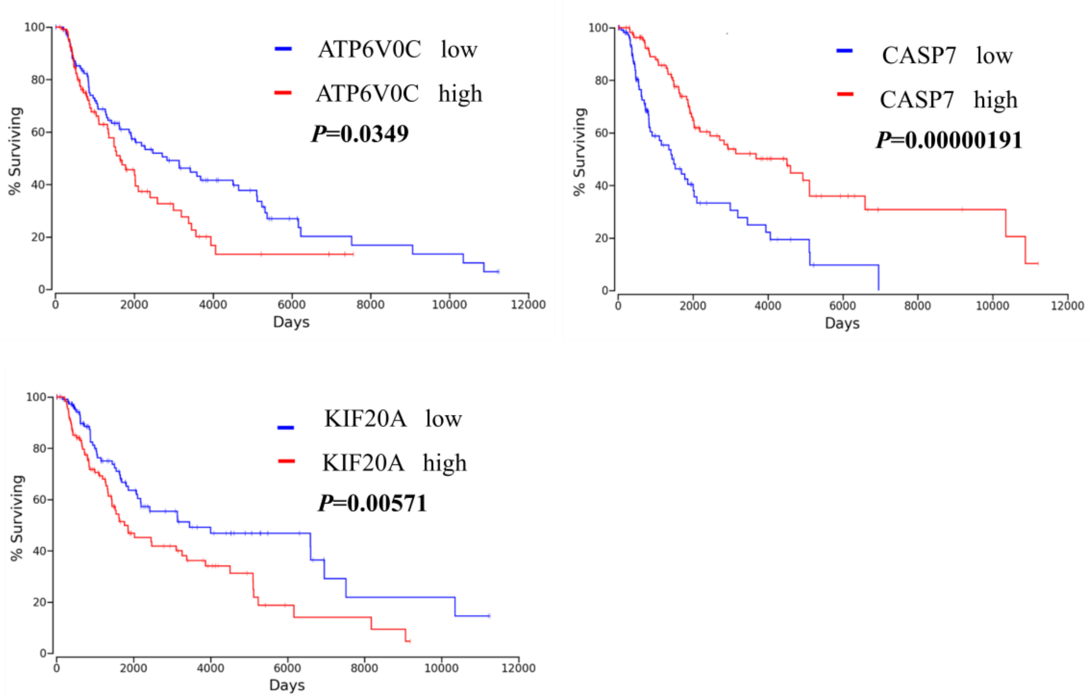

Supplement 6

The western blot analysis of proteins in mice tumors(Fig5.c) were analyzed by ImageJ software. The data shown in the figures are representative of three independent experiments. Scale bar, 50 μm. *:p<0.05,**p<0.01, ***p<0.001.

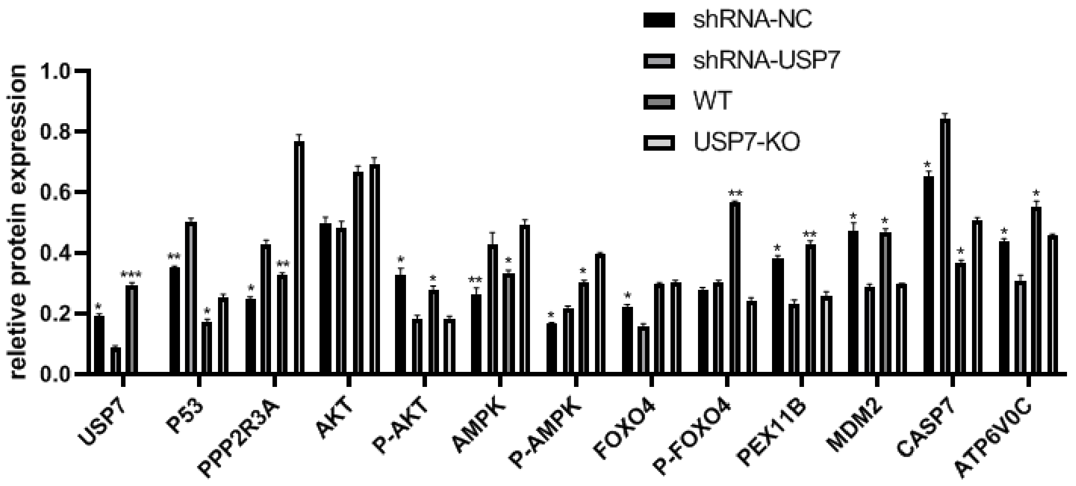

Supplement: Supplementary file 1 [file DataSheet_1.pdf]
